# Bioenergy sorghum stems respond to mechanical stimulation with tissue-specific modifications in hormone homeostasis and anatomical traits

**DOI:** 10.3389/fpls.2026.1769393

**Published:** 2026-03-12

**Authors:** Qing Li, Omid Zargar, Sungkyu Park, Matt Pharr, Anastasia Muliana, Tesfamichael H. Kebrom, Scott A. Finlayson

**Affiliations:** 1 Cooperative Agricultural Research Center, College of Agriculture, Food, and Natural Resources, Prairie View A&M University, Prairie View, TX, United States; 2 Department of Soil and Crop Sciences, Texas A&M University, College Station, TX, United States; 3 Department of Mechanical Engineering, Texas A&M University, College Station, TX, United States

**Keywords:** sorghum bicolor, stem, mechanical stimulation, thigmomorphogenesis, phytohormones, anatomy

## Abstract

Sorghum [*Sorghum bicolor* (L.) Moench] is a promising and highly productive bioenergy crop but remains susceptible to stem lodging, typically caused by severe weather-related mechanical forces like wind and rain. However, when exposed to less severe mechanical stimulation, plants may exhibit alterations in their growth and development through a process known as thigmomorphogenesis that may enhance their ability to withstand stronger forces. Accordingly, understanding mechanisms regulating thigmomorphogenesis may facilitate the development of lodging-resistant sorghum varieties. In this study, the hormonal responses of four stem tissues, including pulvinus, white band, zone of division, and zone of maturation, were investigated at multiple time points following moderate mechanical stimulation, revealing distinct hormonal profiles and tissue-specific response patterns across these tissues. JA level was reduced exclusively in the pulvinus, while GA_1_ level declined specifically in the zone of division. In contrast, the levels of GA_20_, IAA, and ABA decreased in all tissues following mechanical stimulation. Eight weeks of continuous mechanical stimulation reduced plant height and the length of most internodes, primarily by decreasing the number of internodes and reducing cell elongation. Microscopic analysis of internodes at different developmental stages further demonstrated that mechanical stimulation generally increased the density and radial length of pith vascular bundles, enhanced the lignification level in both the pith and rind, and increased the rind thickness. The findings from this study could provide new insights into tissue-specific responses of sorghum stems to mechanical stimulation and may offer new opportunities for improving lodging resistance in sorghum.

## 1 Introduction

Sorghum (*Sorghum bicolor* (L). Moench), a tropical C_4_ grass, has emerged as a promising bioenergy feedstock due to its high nutrient-use efficiency, substantial biomass yield, adaptability to marginal environments, and extensive genetic diversity (Rooney et al., 2007; Mullet et al., 2014). The bioenergy sorghum stem, which constitutes over 80% of the harvested biomass, is the primary determinant of bioenergy yield, with taller stems typically associated with greater biomass accumulation (Olson et al., 2012). However, increased stem height also raises the risk of stem lodging, the bending or breakage of stems. This structural failure is typically caused by severe mechanical forces from weather-related phenomena like wind and rain, leading to significant yield losses, as mechanical harvesters struggle to recover grain or biomass from lodged stems. Additionally, lodging often reduces grain quality by promoting pre-harvest sprouting and increasing susceptibility to molds and fungal infection due to increased exposure to humidity (Berry et al., 2004). Therefore, enhancing stem lodging resistance has become a critical objective in bioenergy sorghum breeding and improvement programs.

Lodging resistance in grain sorghum has been largely improved through the introduction of dwarf genes since the “Green Revolution” (Hedden, 2003). However, the tall stature desirable for high biomass production makes this approach unsuitable for bioenergy sorghum, highlighting the need for alternative strategies to enhance lodging resistance. One potential approach is to enhance the mechanical strength of stems, which depends not only on the geometry of plant organs but also on the material properties of tissues (Niklas, 1992). Sorghum stems are composed of distinct tissues, including the nodal plexus, the pulvinus, the white band, and the internode, which collectively determine the structural integrity (Yu et al., 2022). The nodal plexus, located at the junction where the leaf sheath joins the stem, is enriched in vascular bundles. The pulvinus, positioned above the nodal plexus, serves as a site for nodal root bud formation and participates in hormone signaling. The white band, situated between the pulvinus and internode, potentially facilitates cell division (Yu et al., 2022). The internode, the largest portion of the stem responsible for elongation, is also the primary region where the stem lodging occurs in bioenergy sorghum (Gomez et al., 2017). Within a rapidly elongating internode, the basal region is known as the zone of cell division (ZoD), followed by the zone of elongation (ZoE), and the zone of maturation (ZoM), in which cell proliferation, expansion, and maturation take place, respectively (Oliver et al., 2021). Structurally, the internode consists of the rind (a stiffer outer layer) that includes the epidermis, and the pith (a soft inner core). Both regions contain vascular bundles that provide transport and structural support (Lee et al., 2020). The coordinated development of these tissues determines internode length, thickness, and strength (Kebrom et al., 2017; Yu et al., 2022).

Moderate mechanical stimulation during plant growth and development that does not cause structural damage can induce an adaptive response known as thigmomorphogenesis (Jaffe, 1973). This process involves sustained changes in plant growth and development that enhance the plant’s ability to withstand future mechanical stresses. Historically, farmers in China and Japan have intentionally applied mechanical stimulation, such as stamping or treading, to strengthen crops and reduce lodging under adverse weather conditions (Iida, 2014; Li et al., 2019). These traditional practices are strategically performed at early growth stages when the seedlings are flexible enough to adapt to physical stress without sustaining permanent structural damage. Thigmomorphogenesis is primarily manifested in morphological changes, typically including reduced stem elongation and increased stem diameter (Chehab et al., 2012), which are closely associated with underlying anatomical modifications. For example, the reduced height in mechanically stimulated *Arabidopsis* has been attributed to decreased cell elongation mediated by ethylene signaling (Wu et al., 2020). Other hormones, including jasmonic acid (JA), abscisic acid (ABA), indole-3-acetic acid (IAA), and gibberellins (GA), also play important roles in regulating thigmomorphogenesis (Chehab et al., 2012; Lange and Lange, 2015; Li et al., 2023). Recent evidence further revealed that these hormones interact coordinately to regulate thigmomorphogenesis. In *Arabidopsis*, ethylene and JA signaling have been shown to converge on GA catabolism but play opposite roles during thigmomorphogenesis (Wang et al., 2024). Mechanical stimulation can also alter biochemical composition, such as increasing lignin and cellulose content (De Jaegher et al., 1985; Gladala-Kostarz et al., 2020). These modifications in morphological, anatomical, and biochemical traits alter the biomechanical properties of plants. In sorghum, periodic bending of stems has been shown to reduce the elastic modulus and flexural stiffness while increasing the strength of internodes (Zargar et al., 2022). Additionally, mechanical stimulation can trigger extensive transcriptome reprogramming. In *Arabidopsis*, for example, over 2.5% of the genome shows touch-induced expression, highlighting the significance of thigmomorphogenesis as a fundamental adaptive mechanism (Lee et al., 2005).

Although thigmomorphogenesis has been widely studied in various plant species, the mechanisms underlying thigmomorphogenesis remain incompletely understood, particularly in sorghum. Previous work has shown that mechanical stimulation can increase internode rind thickness, potentially enhancing stem strength (Lemloh et al., 2014). However, its effect on other crucial anatomical traits of sorghum stems, such as vascular bundle size, density, and lignification level, remains poorly understood, and the relationship between these anatomical traits and the observed phenotype of shorter, stronger, and more flexible stems, is still unclear (Zargar et al., 2022). Additionally, prior studies have primarily focused on the internode region of the stem, while the other tissues including nodal plexus, pulvinus and white band have been disregarded, and their hormone profiles remain uncharacterized in sorghum. Given the heterogeneity of these tissues, examining the tissue-specific hormonal response to mechanical stimulation is critical for understanding how distinct stem components contribute differentially to overall stem development and mechanical strength in sorghum. It was hypothesized that distinct stem tissues exhibit differential hormonal and anatomical responses to mechanical stimulation, which collectively contribute to the structural adaptation of sorghum stems. In this study, the hormonal responses of four stem tissues, Pulvinus (PS), White Band (WB), Zone of Division (ZoD), and Zone of Maturation (ZoM), to unidirectional mechanical stimulation at multiple time points were investigated. Anatomical modifications induced by mechanical stimulation were also quantified in two developmentally distinct internodes: internode 4 (older) and internode 9 (younger).

## 2 Materials and methods

### 2.1 Plant material and growth conditions

The sweet sorghum (*Sorghum bicolor* L. Moench) cv. Della was selected for this study, which is an intermediate maturity genotype characterized by reddish-brown seeds and is primarily used for syrup production and bioenergy feedstock. As a high-biomass variety typically growing over 2.5 m tall, it is significantly more vulnerable to weather-related mechanical stresses than dwarf grain sorghums, making it an ideal model for studying thigmomorphogenesis. Characterizing how this cultivar enhances its structural integrity through mechanical adaptation provides valuable insights for improving lodging resistance across diverse sorghum genotypes and other important crops. Seeds were planted on October 27, 2021, in 14.9 L pots (27.6 cm diameter × 28.3 cm height) containing a fine sandy loam soil amended with 28% potting mix (Jolly Gardener C/20). Plants were grown in a greenhouse at Texas A&M University with adequate irrigation and nutrient supply (Osmocote 13-13–13 plus soluble micronutrients). Temperatures were maintained at 26–30 °C during the day and 21–26 °C at night, under a 14 h light/10 h dark photoperiod with supplemental light provided by high-pressure sodium lamps (Lee et al., 2020).

### 2.2 Experimental treatments

**Figure 1:**
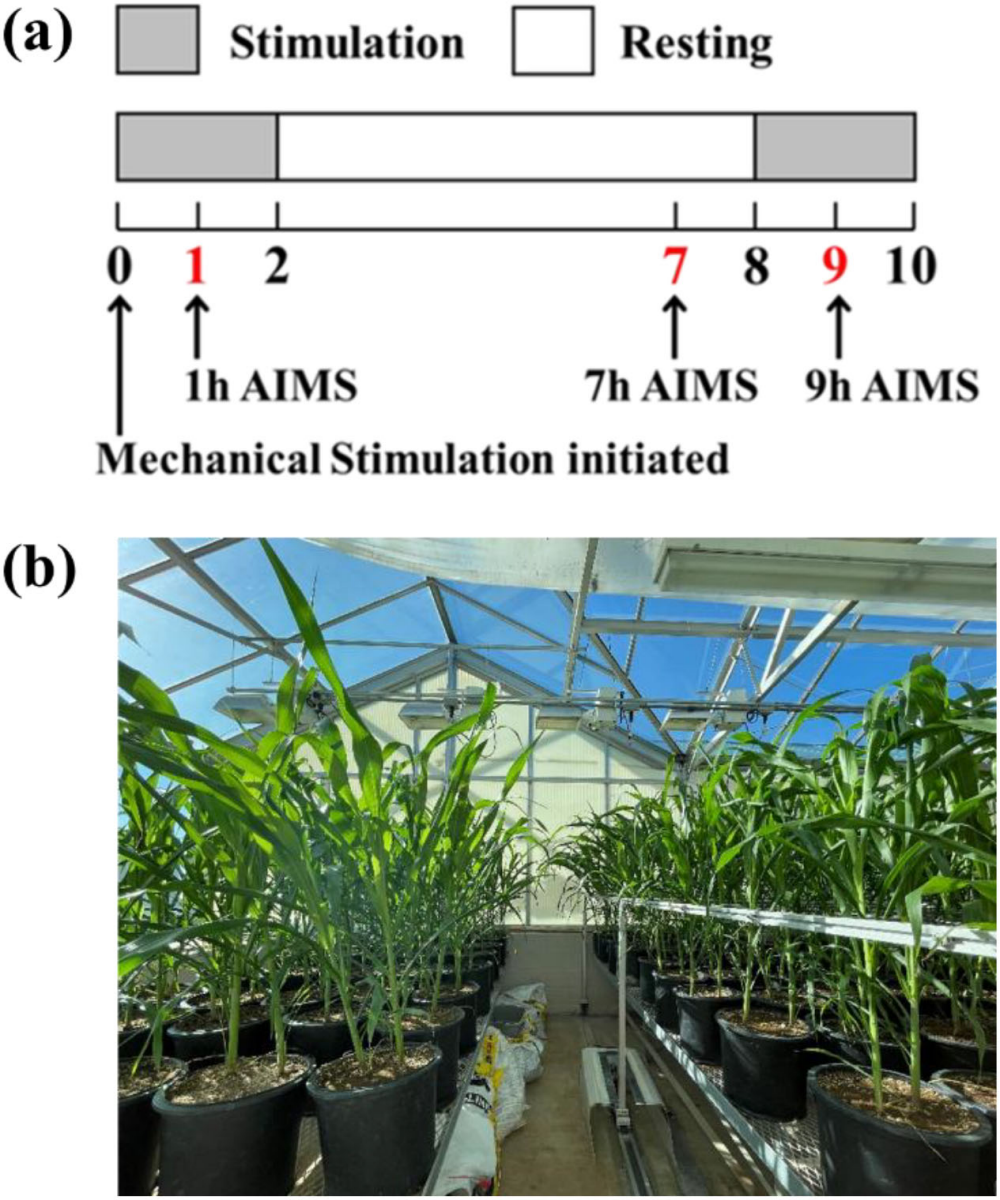
Experimental design. **(a)** Experimental scheme showing the operating cycle of the mechanical apparatus and sampling time points. Stem samples were harvested at 1 h, 7 h, and 9 h After Initiating Mechanical Stimulation (AIMS) as indicated in red. **(b)** Control plants (left) and mechanically stimulated plants (right) in the greenhouse at the onset of stimulation.

Plants were subjected to mechanical stimulation 71 days after sowing, at a growth stage when the sixth internode was typically beginning to elongate and was less than 1 cm long. The mechanical stimulation was applied using a motor-driven rigid frame (Zargar et al., 2022; Li et al., 2023), specifically designed to mimic the natural stem-bending effects like moderate wind or rain. As the frame moved laterally, the rods attached to the frame came into contact with the plants, thereby bending the stems at a frequency of 3 cycles min^-1^ with a 20 cm amplitude continuously for 2 hours, followed by a 6-hour resting period before starting the next stimulation cycle (**Figure 1a**). These parameters were selected to provide a robust mechanical stimulation to trigger thigmomorphogenetic reinforcement while avoiding any potential inertial effects and the risk of structural damage to the stems. The point of stimulation was positioned at 51 cm above the soil surface. An equal number of unstimulated plants were used as controls, which were placed adjacent to the treatment group but without the presence of the rigid frame for mechanical stimulation (**Figure 1b**). For each group, 50 pots were arranged in a 5×10 grid, and their positions were periodically randomized and rotated between and within benches during the growth period prior to the onset of mechanical stimulation.

### 2.3 Sampling methods

**Figure 2:**
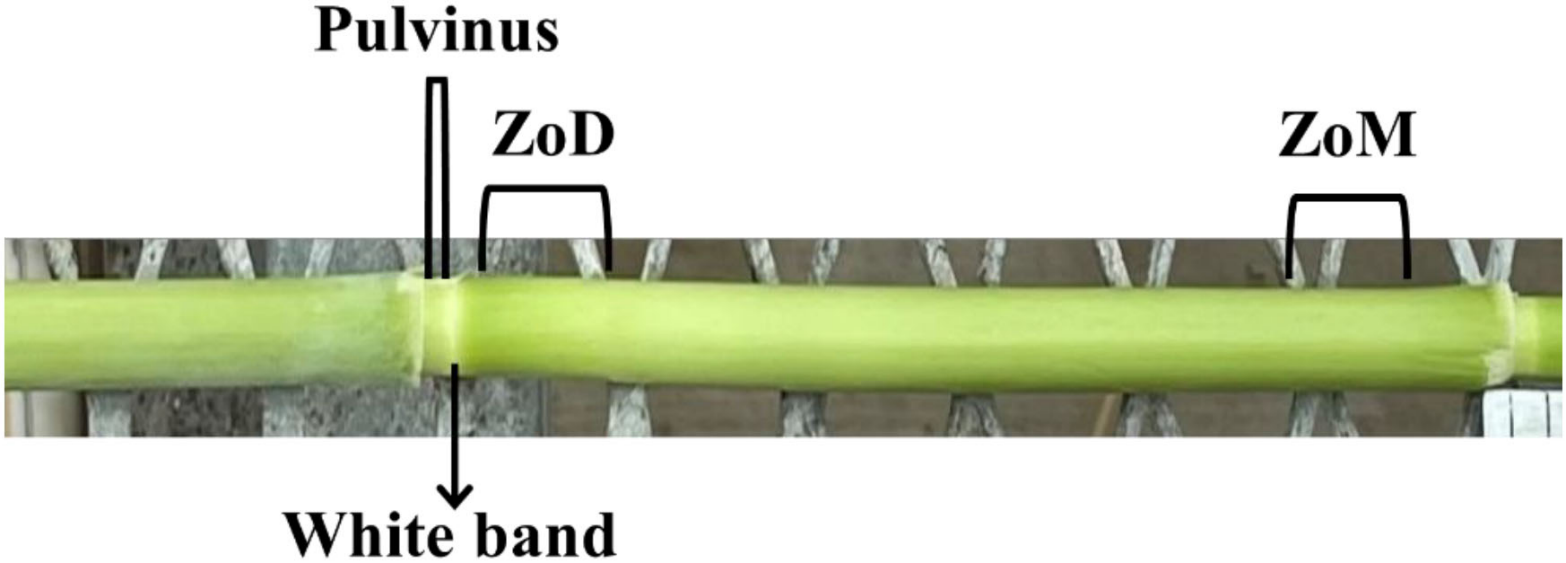
Anatomy of a sorghum stem. Schematic showing the pulvinus, white band, Zone of Division (ZoD) and Zone of Maturation (ZoM) in the longest elongating internode (typically internode 4).

For hormone analysis, stem tissues were sampled from the Pulvinus (PS), White Band (WB), Zone of Division (ZoD), and Zone of Maturation (ZoM) between the two nodal planes flanking on the longest elongating internodes (typically internode 4, numbered from the most basal internode with at least 0.5 cm of elongation) (**Figure 2**). For PS and WB, intact cross-sections of approximately 5 mm and 1 mm thickness, respectively, were harvested. For ZoD and ZoM, only rind tissue was harvested by cutting slices approximately 2 cm long × 5 mm wide × 2 mm deep from the outer edge of the internodes, as preliminary analysis showed stronger hormone signals in the rind compared to the pith, and a prior study reported significant increases in the rind thickness following mechanical stimulation, thereby suggesting that the rind might be a primary site of thigmomorphogenetic response (Lemloh et al., 2014). Each replicate consisted of pooled tissues from six individual plants, with five replicates per treatment/tissue/time point. Samples were collected at 1 h, 7 h, and 9 h After Initiating Mechanical Stimulation (AIMS), and were immediately frozen in liquid N_2_, ground to a fine powder, and stored at -70 °C.

For anatomical analysis, stem cross-sections and longitudinal sections were harvested from the lower part (~ 1 cm above the node) of internode 4 (older, near the end of the elongation phase at the onset of stimulation) and internode 9 (younger, not yet formed at the onset of stimulation) from control and stimulated plants after eight weeks of mechanical stimulation using a small table saw. Samples were fixed overnight in FAA solution (50% ethanol, 5% acetic acid, and 4% formaldehyde), embedded in paraffin, sectioned to 15 µm, and then stained with FASGA, which stains lignified tissues in red and non-lignified or weakly lignified tissues in blue (Tolivia and Tolivia, 1987). Sections were imaged and digitized at 20X magnification using a whole-slide scanner in the Histology Laboratory of the Texas A&M College of Veterinary Medicine & Biomedical Sciences. Three biological replicates per treatment/internode/sectioning orientation were analyzed for anatomical characteristics.

### 2.4 Quantification of JA, ABA, IAA, GA_1_, and GA_20_

Approximately 80 mg of powdered tissue was aliquoted for hormone measurements. Samples were extracted and semi-purified as described by Liu and Finlayson (2019), then derivatized with bromocholine following the method of Kojima et al. (2009). JA, ABA, IAA, GA_20_, and GA_1_ were quantified by LC–MS/MS (Waters Acquity UPLC, Waters TQD) using the appropriate bromocholine derivative precursor/product transitions as described in Park et al. (2024). Stable isotope-labeled standards were included for each hormone. Due to technical limitations, ethylene and cytokinins, which are also known to participate in thigmomorphogenesis, were not quantified in this study.

### 2.5 Image acquisition, processing, and analysis

Whole-slide images of each cross-section and longitudinal section were acquired using a Pannoramic SCAN II slide scanner, which were processed in QuPath (version 0.3.2; Bankhead et al., 2017) to analyze anatomical characteristics, including the cell length, vascular bundle (VB) density, VB size, VB radial length, lignified fraction, and rind thickness. Multiple technical replicates were measured per section and averaged to yield a single representative value for each biological replicate (n = 3 plants) for statistical analysis.

Cell length was calculated as the mean length of 100 intact cells randomly selected and measured from each longitudinal-section image using QuPath.

VB density was determined by counting the vascular bundles (VBs) within three randomly selected equal-sized areas from both the rind (~1.74 mm^2^ each) and pith (~9.67 mm^2^ each) of each cross-section image. These counts were then normalized to the number of VBs per mm^2^ and averaged to obtain the final VB density of the rind and pith in each section. The size and radial length of 20 randomly selected, intact VBs were measured from the pith of each cross-section image. VB size was defined as the total VB area (µm²) and the radial length was measured as shown in **Supplementary Figure 1**. The average of these 20 measurements was used as the representative value for each section. The size and radial length of VBs in the rind were not measured in this study due to their non-uniformity and high variability.

The lignified fraction was quantified from three equal-sized regions from both the pith (~ 2.25 mm^2^) and rind (~ 0.84 mm^2^) of each cross-section image (**Supplementary Figures 2a, d**). The brightness/contrast of the sampling area was enhanced to improve color distinction (**Supplementary Figures 2b, e**), and the LAB color threshold was applied to segment the red-stained area corresponding to lignified tissue (**Supplementary Figures 2c, f**). The size of the segmented red area was then measured, and the lignified fraction was calculated as the ratio of the red area to the total sampling area, multiplied by 100%. This procedure was repeated for all three sampling areas in both rind and pith, and the average lignified fraction was calculated for each tissue type.

To examine the rind thickness, the whole-slide image was imported into QuPath and downsampled (resolution = 30 pixels) before further analysis in Fiji (Schindelin et al., 2012) (**Supplementary Figure 3a**). The brightness/contrast of the image was adjusted to enhance visibility (**Supplementary Figure 3b**), and then it was converted to binary format (**Supplementary Figure 3c**). The QuantiFasga plugin (Legland et al., 2017) was used to segment the rind of the stem cross section (**Supplementary Figure 3d**). Rind thickness was calculated as the average of eight measurements taken at distinct positions of the rind, avoiding any distorted edges based on the original image.

### 2.6 Statistical analysis

Unpaired t-tests were conducted in R (version 4.1.1) to compare plant height, number of internodes, flowering time, anatomical characteristics, and hormone abundances between mechanically stimulated groups and controls. Differences in morphological traits of all internodes between control and stimulated groups, as well as differences in hormone levels among the various tissue types within the stem, were evaluated using two-way analysis of variance (ANOVA). Assumptions of normality and the homogeneity of variance were assessed using Shapiro-Wilk test and Levene’s test, respectively. The *post-hoc* pairwise comparisons following significant ANOVA results were performed using the “emmeans” package in R, with p-values adjusted with the Tukey HSD method to control for multiple comparisons.

## 3 Results

### 3.1 Tissue-specific phytohormone distribution in control stems

**Figure 3:**
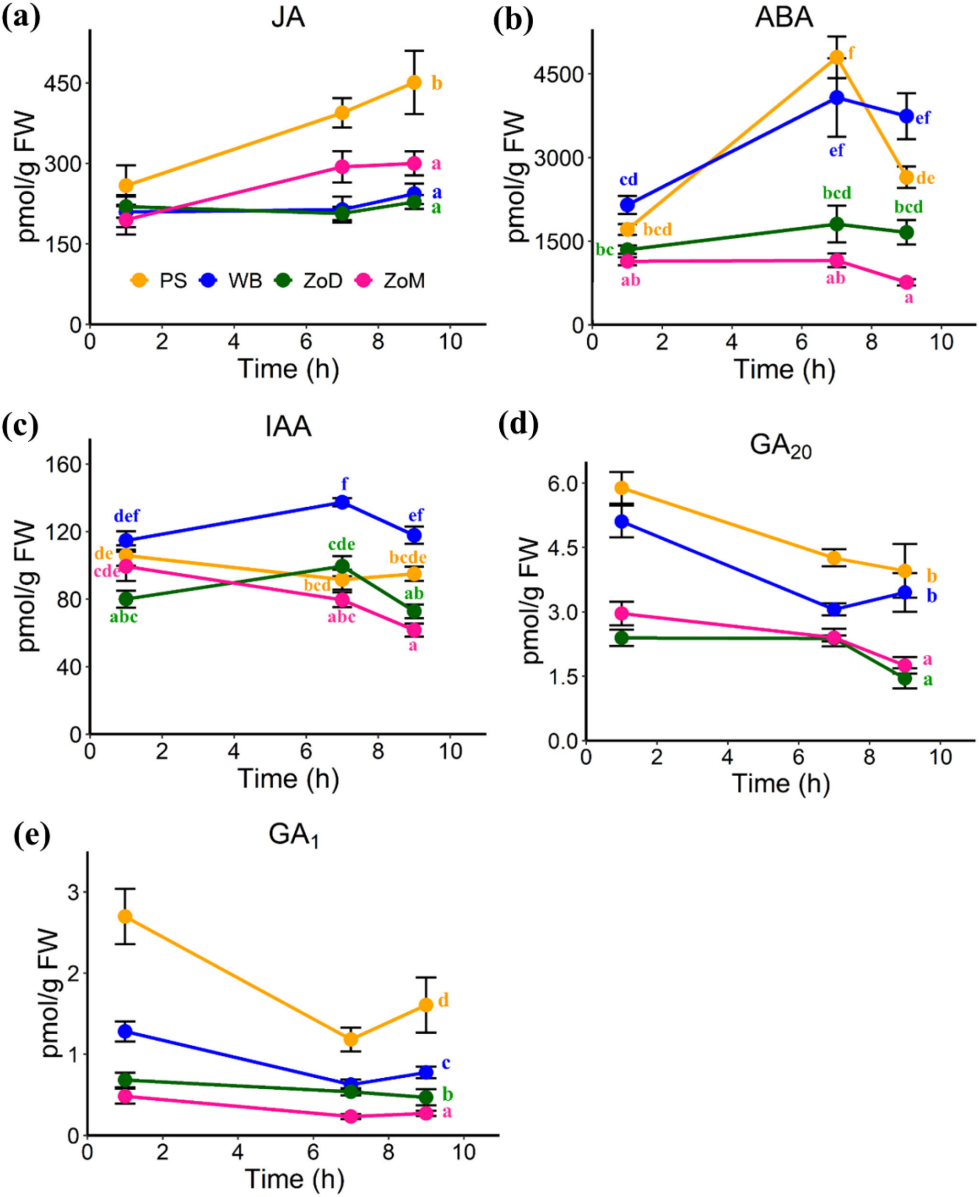
Plant hormone abundances in the Pulvinus (PS), White Band (WB), Zone of Division (ZoD), and Zone of Maturation (ZoM) of control stems at 9:00 a.m., 3:00 p.m., and 5:00 p.m. (corresponding to 1 h, 7 h, and 9 h AIMS in stimulated plants). Tissue types are distinguished by color: orange = PS, blue = WB, green = ZoD, red = ZoM. **(a)** Jasmonic acid (JA); **(b)** Abscisic acid (ABA); **(c)** Indole-3-acetic acid (IAA); **(d)** Gibberellin A_20_ (GA_20_); **(e)** Gibberellin A_1_ (GA_1_). Data are means ± SE (n = 5) analyzed using two-way ANOVA. For JA **(a)**, GA_20_**(d)**, and GA_1_**(e)**, no interaction between tissue and time was detected, so a single letter was used to indicate the significant differences among various tissue types. For ABA **(b)** and IAA **(c)**, a significant interaction between tissue and time was detected, so each tissue-time combination was labeled separately with letters to indicate significant differences (*p*-value < 0.05).

Phytohormone abundances were analyzed in the PS, WB, ZoD, and ZoM of control stems harvested at three different time points (**Figure 2**), corresponding to 1 h, 7 h, and 9 h After Initiating Mechanical Stimulation (AIMS) in stimulated plants (9:00 a.m., 3:00 p.m., and 5:00 p.m., respectively), which may provide insights into the roles of these tissues in stem morphogenesis. Two-way ANOVA for JA showed a significant main effect of tissue but no tissue × time interaction (**Supplementary Table 1.1**). Multiple comparisons revealed that the JA level was significantly higher in PS than in WB, ZoD, and ZoM, while no significant differences were detected among WB, ZoD, and ZoM (**Figure 3a**). For both ABA and IAA, a significant tissue × time interaction was detected (**Supplementary Tables 1.2, 1.3**), indicating that the abundances of ABA and IAA varied depending on the specific tissue-time combinations. At 1 h AIMS (~ 9:00 a.m.), the ABA level was relatively similar in all four tissues. However, at 7 h AIMS (~ 3:00 p.m.), the ABA level in PS and WB was significantly higher than that in ZoD and ZoM. By 9 h AIMS (~ 5:00 p.m.), the ABA level in PS was significantly decreased, while no significant change was observed in the other three tissues (**Figure 3b**). The IAA level was similar in the four tissues at 1 h and 9 h AIMS, but at 7 h AIMS, WB showed significantly higher IAA abundance than PS, ZoD, and ZoM (**Figure 3c**). Two-way ANOVA for GA_20_ and GA_1_ indicated significant main effects of tissue but without tissue × time interactions (**Supplementary Tables 1.4, 1.5**). Multiple comparisons showed no significant difference in GA_20_ level between the PS and WB, or between the ZoD and ZoM. However, the GA_20_ level was significantly higher in the PS and WB compared to the ZoD and ZoM (**Figure 3d**). The GA_1_ abundance followed a decreasing trend across the 4 tissues, with PS > WB > ZoD > ZoM (**Figure 3e**).

### 3.2 Tissue-specific phytohormone responses of stems to mechanical stimulation

**Figure 4:**
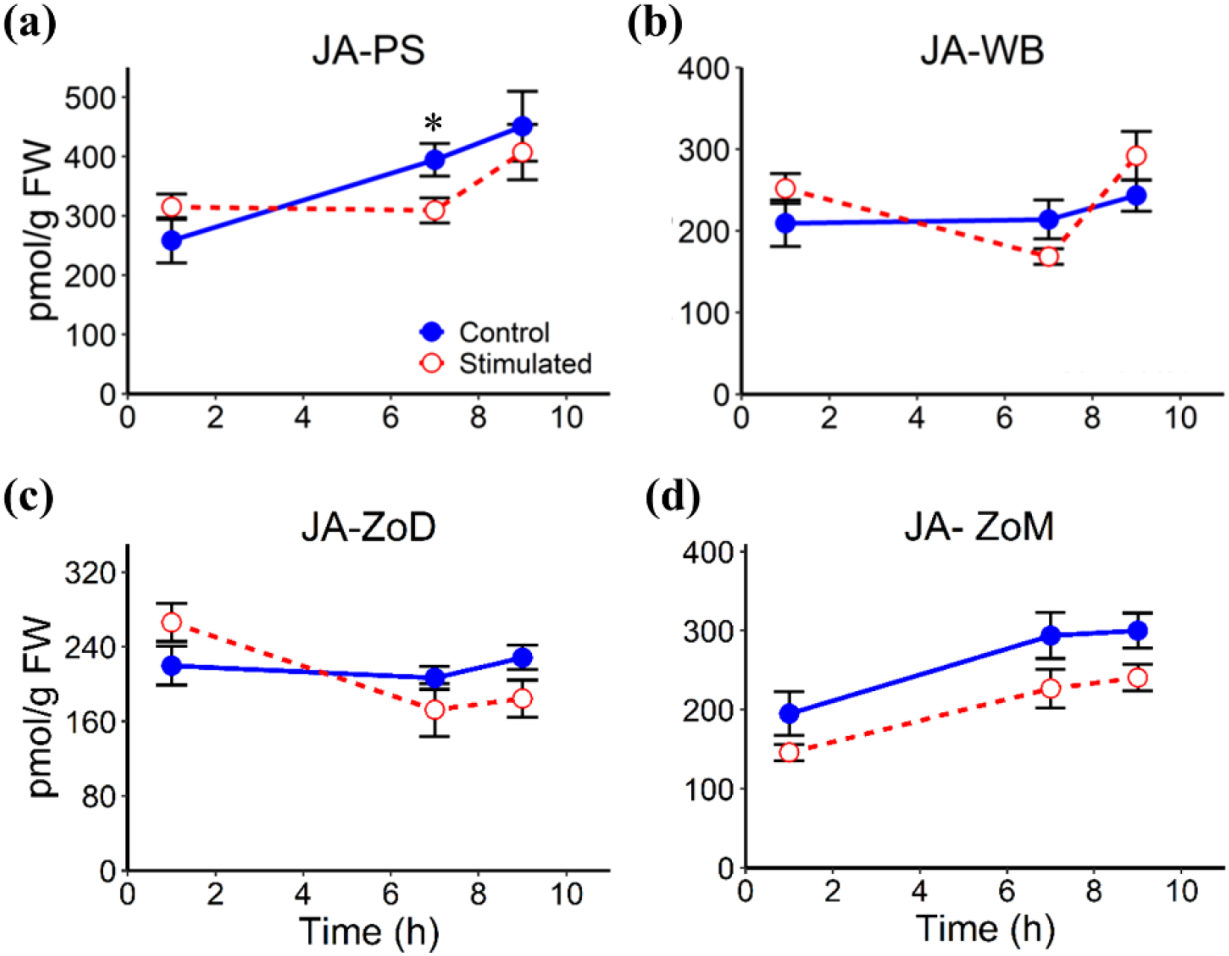
JA levels in stem tissues at 1 h, 7 h, and 9 h AIMS. **(a)** Pulvinus (PS); **(b)** White Band (WB); **(c)** Zone of Division (ZoD); **(d)** Zone of Maturation (ZoM). Data are means ± SE (n = 5). Asterisks (*) indicate significant differences (*p*-value < 0.05) between control and stimulated tissues at the same time point.

**Figure 5:**
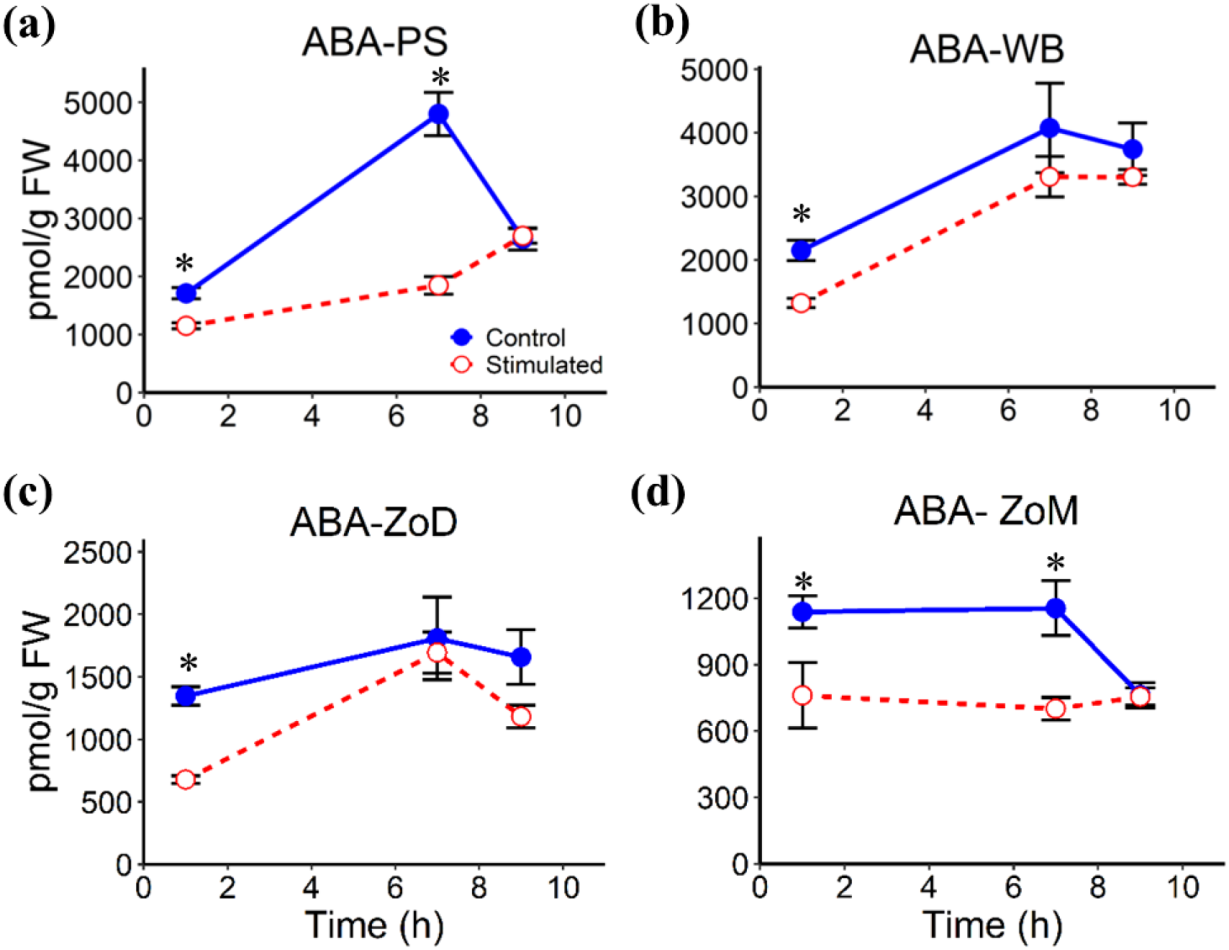
ABA levels in stem tissues at 1 h, 7 h, and 9 h AIMS. **(a)** Pulvinus (PS); **(b)** White Band (WB); **(c)** Zone of Division (ZoD); **(d)** Zone of Maturation (ZoM). Data are means ± SE (n = 5). Asterisks (*) indicate significant differences (*p*-value < 0.05) between control and stimulated tissues at the same time point.

**Figure 6:**
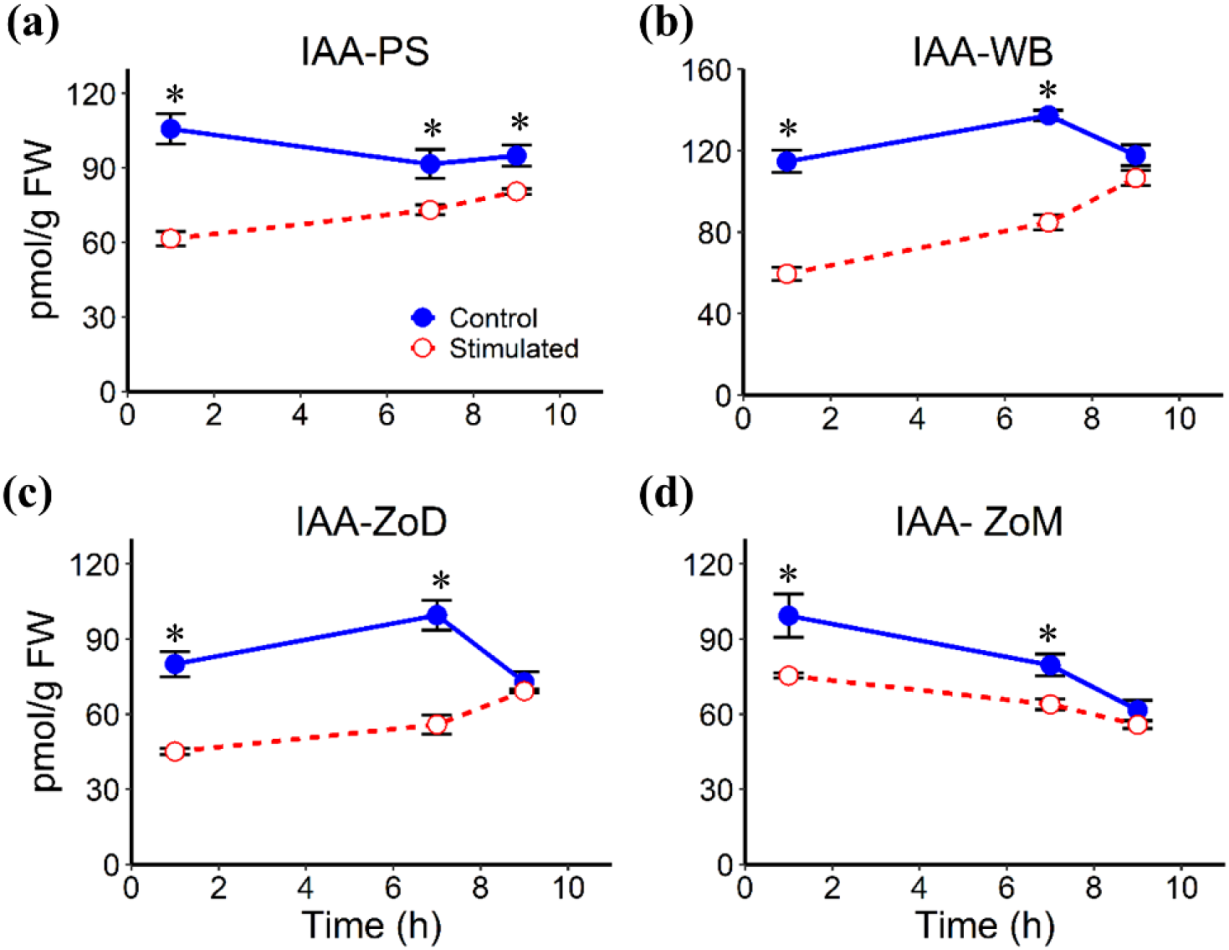
IAA levels in stem tissues at 1 h, 7 h, and 9 h after AIMS. **(a)** Pulvinus (PS); **(b)** White Band (WB); **(c)** Zone of Division (ZoD); **(d)** Zone of Maturation (ZoM). Data are means ± SE (n = 5). Asterisks (*) indicate significant differences (*p*-value < 0.05) between control and stimulated tissues at the same time point.

**Figure 7:**
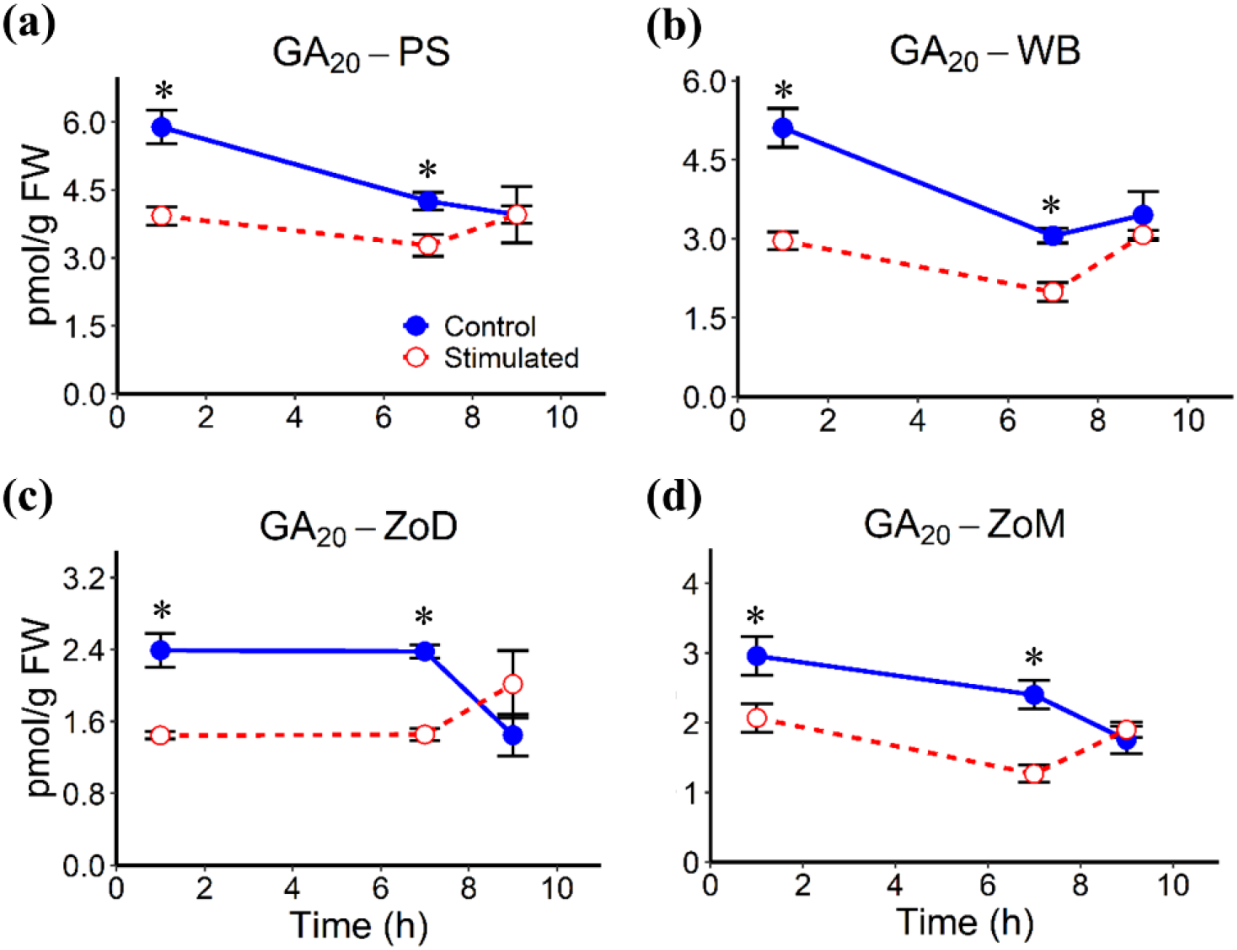
GA_20_ levels in stem tissues at 1 h, 7 h, and 9 h AIMS. **(a)** Pulvinus (PS); **(b)** White Band (WB); **(c)** Zone of Division (ZoD); **(d)** Zone of Maturation (ZoM). Data are means ± SE (n = 5). Asterisks (*) indicate significant differences (*p*-value < 0.05) between control and stimulated tissues at the same time point.

**Figure 8:**
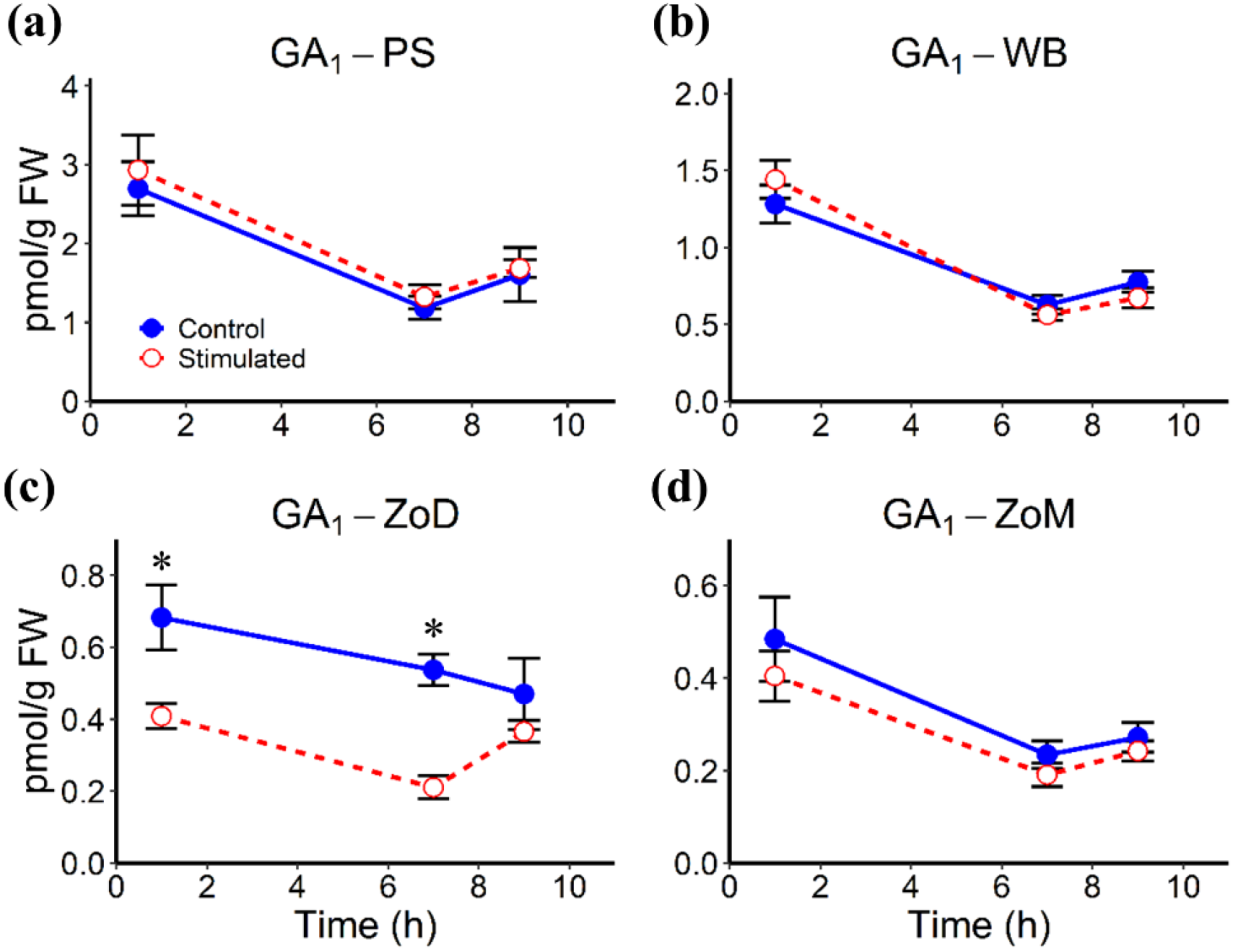
GA_1_ levels in stem tissues at 1 h, 7 h, and 9 h AIMS. **(a)** Pulvinus (PS); **(b)** White Band (WB); **(c)** Zone of Division (ZoD); **(d)** Zone of Maturation (ZoM). Data are means ± SE (n = 5). sterisks (*) indicate significant differences (*p*-value < 0.05) between control and stimulated tissues at the same time point.

Mechanical stimulation altered the abundances of several phytohormones in stem tissues, including the White Band (WB), Pulvinus (PS), Zone of Division (ZoD), and Zone of Maturation (ZoM), at 1 h, 7 h, and 9 h AIMS, corresponding to 1 h after the first stimulation cycle, 1 h before the second cycle, and 1 h after the second cycle, respectively. JA levels remained largely unchanged in all tissues, except for a significant decrease in the PS at 7 h AIMS (**Figure 4**). ABA abundance was significantly reduced at 1 h and 7 h AIMS in the PS and ZoM, but only at 1 h AIMS in the WB and ZoD (**Figure 5**). IAA levels were suppressed by mechanical stimulation at 1 h and 7 h AIMS in all four tissues, and were also lower than controls in the PS at 9 h AIMS (**Figure 6**). Similarly, GA_20_, the immediate precursor of bioactive GA_1_, declined at 1 h and 7 h AIMS in all four tissues (**Figure 7**). GA_1_ levels in mechanically stimulated plants were comparable to controls in PS, WB, and ZoM, but were significantly reduced in ZoD at 1 h and 7 h AIMS (**Figure 8**).

### 3.3 Stem morphological traits in control and mechanically stimulated plants

**Figure 9:**
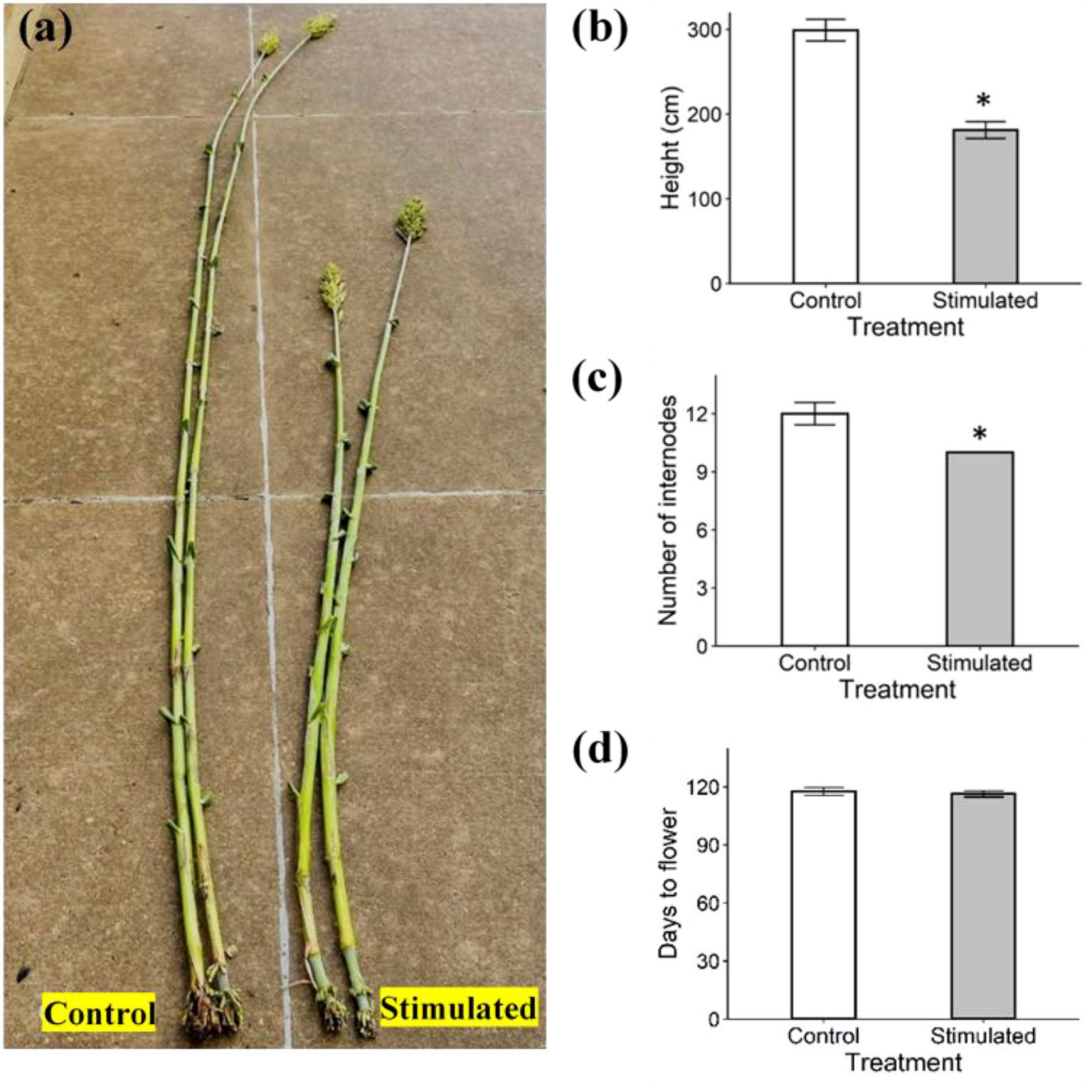
Control and stimulated plants after eight weeks of mechanical stimulation treatment (127 days after planting). **(a)** Control (left) and stimulated (right) plants. **(b)** Plant height. **(c)** Total number of internodes. **(d)** Days to flower after planting. Data are means ± SE (n = 3 for plant height and internode number, n = 21 for flowering time). Asterisks (*) indicate significant differences (*p*-value < 0.05) between control and stimulated plants.

**Figure 10:**
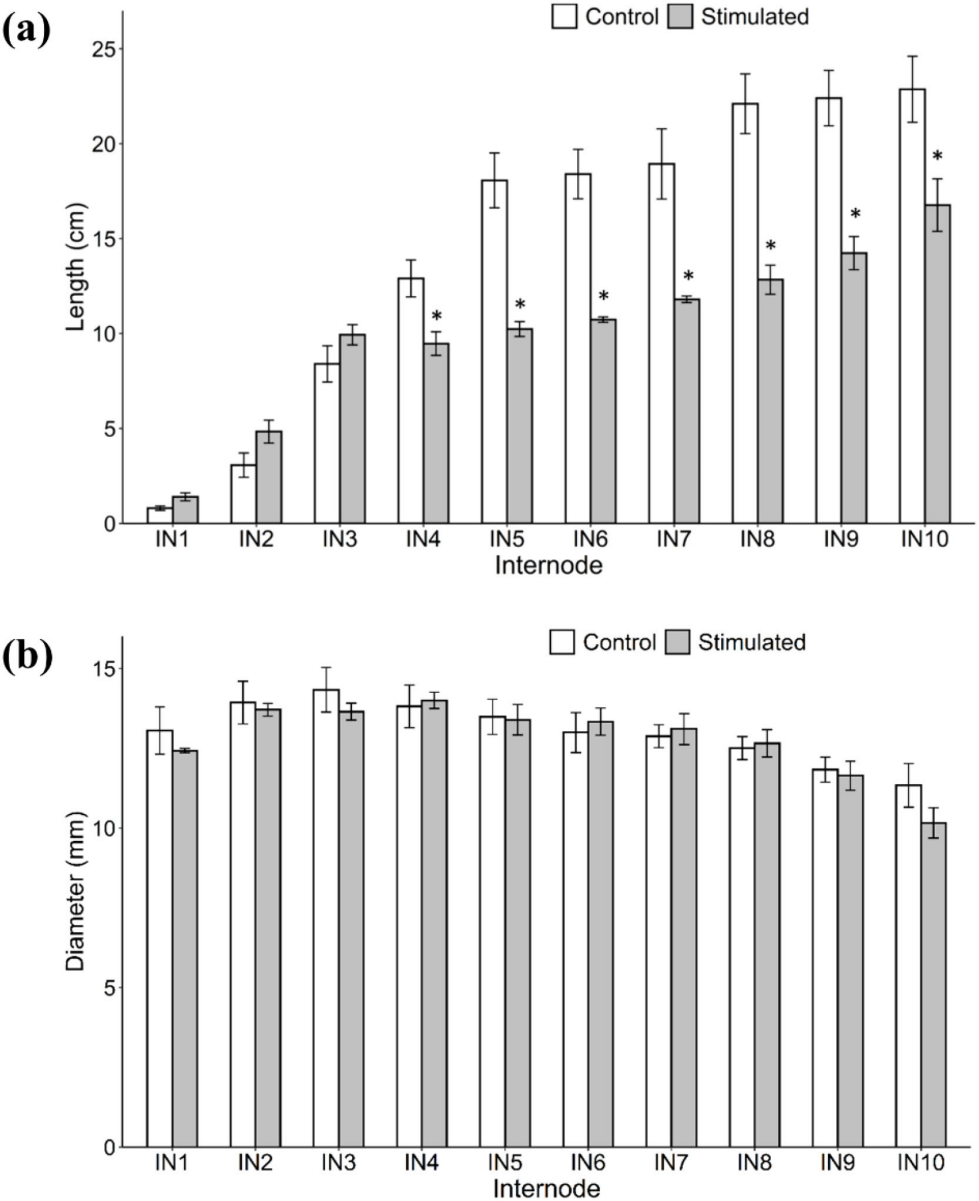
Internode traits of control and stimulated plants after eight weeks of mechanical stimulation treatment. **(a)** Length of the first 10 internodes (IN1 – IN10). **(b)** Diameter of the first 10 internodes (measured at ~ 1 cm above the node). Data are means ± SE (n = 3). Asterisks (*) indicate significant differences (*p*-value < 0.05) between control and stimulated internodes.

Several morphological traits were evaluated in control and mechanically stimulated plants, including the plant height, number of internodes, and the internode length and diameter. At harvest (eight weeks after initiating mechanical stimulation), stimulated plants were significantly shorter than control plants, reaching an average height of 181 cm compared to 299 cm for the control group (**Figures 9a, b**), and also produced fewer internodes (**Figure 9c**). In contrast, the flowering time did not differ significantly between control and stimulated plants (**Figure 9d**). Two-way ANOVA was used to compare the length and diameter of the first ten internodes between two groups (**Supplementary Table 2**). Mechanical stimulation significantly reduced the lengths of all internodes except internode 1, 2, and 3 (**Figure 10a**). However, no significant differences were detected in the internode diameter (measured ~ 1 cm above the node) between the control and stimulated groups (**Figure 10b**).

### 3.4 Stem anatomical characteristics in control and mechanically stimulated plants

**Figure 11:**
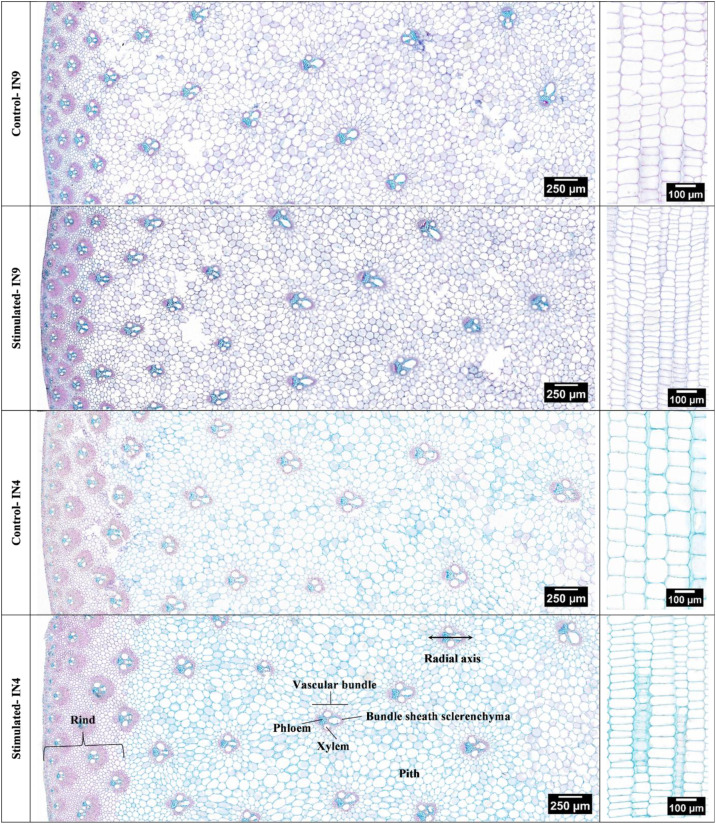
Representative images of FASGA-stained cross- and longitudinal sections for internode 4 (IN4) and internode 9 (IN9) from control and mechanically stimulated plants. Red staining indicates lignin accumulation.

**Figure 12:**
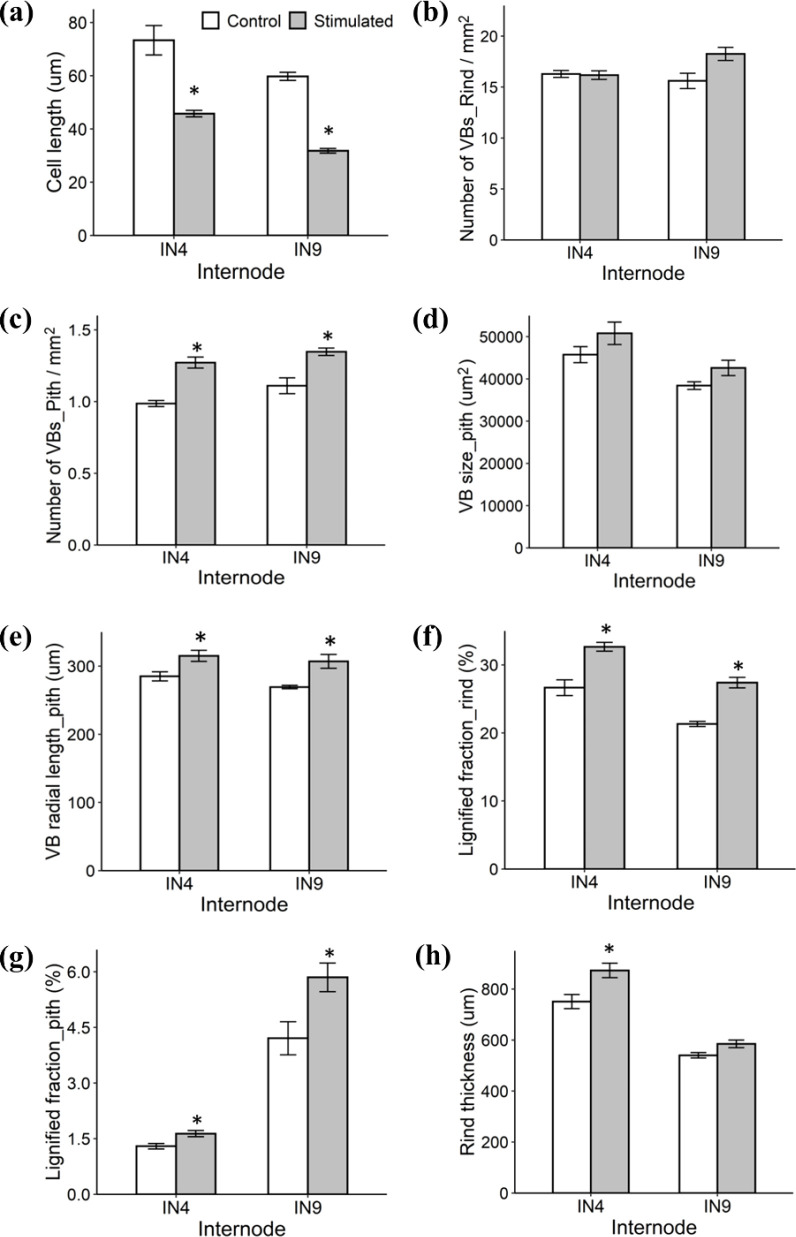
Anatomical traits of internode 4 (IN4) and internode 9 (IN9) from control and stimulated plants. **(a)** Cell length. **(b)** Vascular bundle **(VB)** density in the rind. **(c)** VB density in the pith. **(d)** VB size in the pith. **(e)** VB radial length in the pith. **(f)** Lignified fraction in the rind. **(g)** Lignified fraction in the pith. **(h)** Rind thickness. Data are means ± SE. Asterisks (*) indicate significant differences (*p*-value < 0.05) between control and stimulated internodes.

Mechanical stimulation-induced morphological changes are closely associated with alterations in stem anatomy, as previously reported (Biro et al., 1980; Schoelynck et al., 2015; Gladala-Kostarz et al., 2020). Representative FASGA-stained cross- and longitudinal sections of internodes 4 (older, near the end of the elongation phase at the onset of mechanical stimulation) and 9 (younger, not yet formed at the onset of mechanical stimulation) are shown in **Figure 11**. Longitudinal sections revealed that cell length was significantly reduced in stimulated plants for both internodes (**Figure 12a**), which is consistent with the reduced plant height observed after mechanical stimulation (**Figures 9a, b**). Cross-sections showed no significant differences in Vascular Bundle (VB) density in the rind (**Figure 12b**), but VB density in the pith was significantly increased in both stimulated internodes 4 and 9 (**Figure 12c**). Although VB size in the pith did not differ significantly between control and stimulated internodes (**Figure 12d**), mechanical stimulation altered the VB shape, resulting in significantly greater radial elongation (**Figure 12e**). Mechanical stimulation also significantly increased lignification in both rind and pith of both internodes (**Figures 12f, g**). Additionally, the rind thickness was significantly increased in stimulated internode 4 compared to the control (**Figure 12h**).

## 4 Discussion

### 4.1 Mechanical stimulation alters morphological and anatomical traits of sorghum stems

The present study demonstrated that eight weeks of mechanical stimulation significantly reduced plant height and the length of most internodes in sorghum, consistent with the findings of Zargar et al. (2022). The decrease in plant height likely resulted from reduced internode cell elongation and fewer internodes, which may be associated with decreased levels of the IAA and GA_1_ hormones known to promote stem elongation (Campanoni and Nick, 2005; Kou et al., 2021). Interestingly, while Zargar et al. (2022) observed a modest increase in the diameter of the middle part of internodes following mechanical stimulation, no significant changes in diameter were detected in the lower part of internodes in this study.

Mechanical stimulation also altered stem anatomy. These changes were examined through microscopic analysis of two internodes at different developmental stages: internode 4 (older, near the end of the elongation phase at the onset of mechanical stimulation) and internode 9 (younger, not yet formed at the onset of mechanical stimulation). Both internodes exhibited an increase in the Vascular Bundles (VB) density in the pith following mechanical stimulation, while VB density in the rind remained unchanged. A prior study has linked higher VB density to increased stem bending resistance in wheat (Wang et al., 2006), suggesting that the increased VB density observed here may contribute to enhanced stem strength in sorghum, consistent with the earlier report (Zargar et al., 2022). Although the VB size in the pith did not differ between control and stimulated internodes, mechanical stimulation altered the VB geometry, resulting in greater radial elongation. This change may serve as an adaptive strategy to strengthen internodes against external mechanical forces. Additionally, mechanical stimulation increased the lignin levels in both the rind and the pith of both stimulated internodes. The enhanced lignification in the pith may be associated with increased pith VB density, as cells surrounding vascular bundles are known to accumulate greater amounts of lignin and cellulose. Transcriptome analysis of sorghum stems has also highlighted the importance of lignin in response to mechanical stimulation (Li et al., 2023), and an increase in lignin content has been reported in *Brachypodium distachyon* subjected to wind treatment (Gladala-Kostarz et al., 2020). Evidence from rice further underscores the importance of lignin. For example, loss of CESA4 reduces cellulose, hemicellulose, and lignin levels, leading to weaker culms (Ma et al., 2021), which suggests that increased lignin content may enhance the mechanical strength of stems, consistent with previous reports in wheat (Kong et al., 2013). Mechanical stimulation also increased the rind thickness in internode 4, consistent with prior observation of thickened rinds in sorghum subjected to periodic mechanical stress (Lemloh et al., 2014). Interestingly, the rind thickness in internode 9 remained unaffected. This discrepancy may be associated with the larger and longer mechanical stress experienced by internode 4 due to its lower position within the stem and its earlier development. Interestingly, although the rind VB density remained unchanged, lignification and rind thickness increased following mechanical stimulation. This suggest the VB patterning, which is established early during stem development (Scarpella and Meijer, 2004), may remain developmentally constrained in the rind, while the lignification, occurring later during secondary cell wall deposition after cell elongation has ceased, exhibits greater plasticity in response to mechanical stimulation. The increased rind thickness likely reflects enhanced secondary cell wall deposition extending further into adjacent ground tissues.

### 4.2 Tissue-specific variations in hormone levels within the sorghum stem

Hormone signaling is a central regulatory mechanism in stem elongation (Multani et al., 2003; Heinrich et al., 2013). However, how different hormones function within distinct stem tissues remains poorly understood. This study sheds light on the variations in hormone distribution across four tissues of the sorghum stem, including the Pulvinus (PS), White Band (WB), Zone of Division (ZoD), and Zone of Maturation (ZoM). JA has been primarily associated with plant defense responses against pathogens and pests (Wasternack and Hause, 2013). The JA level was consistently higher in the PS than in the WB, ZoD, and ZoM at all three time points, indicating a potential role of JA in repressing adventitious root formation in the PS (Lakehal et al., 2020). Additionally, gibberellins (GAs) are well known to promote stem elongation (Kou et al., 2021). In this study, the bioactive GA_1_ level showed a clear tissue-specific distribution, with the highest level in the PS, followed by the WB, ZoD, and ZoM. As expected, the GA_1_ level was higher in the WB and ZoD than in the ZoM, as these regions were closely associated with cell division and elongation (Yu et al., 2022). The ZoM exhibited the lowest GA_1_ level, consistent with the cessation of cell elongation and the development of secondary cell walls in this zone. Unexpectedly, the GA_1_ level was most abundant in the PS, despite previous findings indicating that cell division was not observed in the PS (Yu et al., 2022). However, considering that Yu et al. (2022) reported that the pulvinus had not elongated within a four-day period, it is possible that the PS elongation occurs at a later stage, consistent with our observation of increased PS length when the internode was fully elongated. IAA, a principal regulator of plant growth and development, plays essential roles in cell division and cell elongation (Campanoni and Nick, 2005), while ABA functions as a key regulator of abiotic stress responses such as drought and salinity (Zhang et al., 2006). IAA and ABA levels varied among the four tissues over the course of the day, potentially reflecting diurnal fluctuations (Nováková et al., 2005). Overall, the WB contained a higher IAA abundance than the other three tissues, consistent with the established role of IAA in promoting cell division, a process closely associated with the WB (Yu et al., 2022). Surprisingly, the ABA level was higher in the PS and WB, especially at 7 h AIMS (3:00 p.m.), compared to the ZoD and ZoM. This pattern was similar to that of GA_20_ and GA_1_, which were also higher in PS and WB. Although this seems contradictory to the typical role of ABA as an antagonist of gibberellin-mediated stem elongation (Shu et al., 2018), the higher ABA levels in these regions may indicate a role of ABA in fine-tuning growth by limiting excessive elongation during stem development. Further studies are needed to elucidate the mechanisms underlying these tissue-specific hormone distributions and their functional significance in regulating stem elongation.

### 4.3 Tissue-specific hormonal responses to mechanical stimulation within the sorghum stem

Hormonal signaling is a key component of thigmomorphogenesis in many plant species, including sorghum (Chehab et al., 2009; Li et al., 2023). Although previous studies have examined the effect of mechanical stimulation on hormone homeostasis in the elongating zone of the internodes (Li et al., 2023), little is known about how distinct tissue types within the stem respond to mechanical stimulation at the hormone level. In this study, the alterations in hormone homeostasis across four distinct stem tissues in response to mechanical stimulation were investigated.

Mechanical stimulation significantly reduced JA levels specifically in the PS at 7 h AIMS (After Initiating Mechanical Stimulation), during the resting period of the stimulation apparatus, which contrasts with the JA accumulation typically observed following mechanical stimulation. In *Arabidopsis*, JA signaling is a primary driver of thigmomorphogenesis, as evidenced by the touch-insensitivity of JA-deficient *aos* mutants and the constitutive thigmomorphogenic phenotype of *OPR3* transgenic overexpression lines (Chehab et al., 2012). Similarly, elevated JA levels were also detected in *Medicago truncatula* (Tretner et al., 2008) and even within the Zone of Elongation (ZoE) of older internodes in sorghum (Li et al., 2023) following mechanical stimulation. The divergence of the PS response suggests a distinct role of JA within the specific stem tissue. The PS is a critical site for nodal root bud formation (Yu et al., 2022). Given that JA is a known inhibitor of adventitious root initiation (Dob et al., 2021), this localized reduction of JA in the PS may act to alleviate the inhibitory constraints, potentially facilitating root development as a secondary anchorage strategy to enhance plant stability. In *Brachypodium distachyon*, the adventitious root formation was observed following wind treatment (Nam et al., 2020). These findings suggest that JA-mediated thigmomorphogenesis is both species- and tissue-specific, reflecting the complexity and fine-tuned nature of plant hormonal regulation in response to mechanical stimulation.

ABA levels in the WB and ZoD were significantly decreased at 1 h AIMS, while in the PS and ZoM, the decline persisted at both 1 h and 7 h AIMS, indicating parallel responses within these tissue pairs. The similar decrease pattern of ABA in WB and ZoD may be associated with their spatial proximity and previously reported transcriptomic similarity (Yu et al., 2022), while the comparable decline in the spatially distant PS and ZoM was more surprising. However, Yu et al. (2022) also found both PS and ZoM had strong enrichment in secondary cell wall biogenesis and lignin metabolism, indicating some similarities between the two regions despite their separation in the internode. The ABA reduction in the four tissues in this study was consistent with our earlier observations in the ZoE of sorghum internodes following mechanical stimulation (Li et al., 2023), suggesting a homogeneous hormonal response of distinct stem tissues to mechanical stimulation in sorghum. However, the observed decline in ABA contrasts with earlier studies that reported increased ABA level following mechanical stimulation (Jeong and Ota, 1980; Erner and Jaffe, 1982). This discrepancy may be attributable to both species-specific variations and the duration of the stimulation. The historical studies implemented prolonged stimulation for over 10 to 30 days before hormone measurement in rice and bean, while our study captured the response within hours of treatment in sorghum. Although the specific function of ABA in thigmomorphogenesis remains unclear, its reduction in *Arabidopsis* has been linked to reduced cell wall stiffness and a corresponding increase in flexibility, thus highlighting the key role of ABA in maintaining the cell wall’s mechanical properties (Bacete et al., 2022). This finding may also account for the enhanced flexibility of sorghum stems following mechanical stimulation, which was associated with reduced ABA level (Zargar et al., 2022; Li et al., 2023). Further studies are needed to elucidate how ABA contributes to the modulation of cell wall mechanics during thigmomorphogenesis.

GA_1_ and IAA are well known to promote stem elongation (Kou et al., 2021) and have also been implicated in thigmomorphogenesis. An early study in *Phaseolus vulgaris* demonstrated that mechanical perturbation decreased stem elongation and bioactive GA levels (Suge, 1978). Similarly, repetitive touching in *Arabidopsis* reduced bolt height and the bioactive GA_4_ (Lange and Lange, 2015). In this study, mechanical stimulation significantly reduced the level of the GA_1_ precursor, GA_20_, at 1 h and 7 h AIMS across all four stem tissues. However, this decrease was not accompanied by a corresponding decline in GA_1_ levels in the PS, WB, or ZoM, unlike in the ZoD where both GA_1_ and GA_20_ levels were reduced, suggesting tissue-specific regulation of GA_1_ biosynthesis and metabolism. Reduced GA_1_ and GA_20_ levels were also detected in the ZoE of sorghum internodes (Li et al., 2023), showing a similar pattern to the ZoD. GAs are well known to promote stem elongation by inducing the degradation of growth-repressing DELLA proteins (Achard et al., 2009). Therefore, this decrease of bioactive GAs likely results in DELLA accumulation, which restricts both cell expansion and proliferation, ultimately leading to the reduced cell length and decreased total height of mechanically stimulated plants. Additionally, inhibition of GA biosynthesis using uniconazole (S3307) in *Camellia oleifera* significantly upregulated the lignification-related genes and increased the lignin and cellulose accumulation (Zhang et al., 2025), which supports a link between reduced GA level and enhanced lignification following mechanical stimulation.

Similarly, mechanical stimulation also decreased IAA levels at 1 h and 7 h AIMS across all four stem tissues, with an additional decline in the PS at 9 h AIMS, which was consistent with the reduced IAA levels in the ZoE of sorghum internodes reported before (Li et al., 2023). Given that sorghum internode elongation relies on the sufficient supply of auxin via polar transport stream (Multani et al., 2003), this reduction in IAA level represents a significant physiological constraint on vertical growth. The reduced IAA level likely suppresses TMK (Transmembrane Kinase) receptor activity, thereby resulting in a reduction in cell-wall acidification, which hinders cell-wall loosening and expansion, directly contributing to the observed decrease in cell elongation (Lin et al., 2021). Although the wind-induced adventitious root formation in *Brachypodium distachyon* has also been associated with auxin signaling, the effect was attributed to soil contact with the stem rather than stem bending stresses (Nam et al., 2020). In view of the fundamental role of GA and IAA in plant architecture, further investigation into how these hormones regulate thigmomorphogenesis seems warranted.

### 4.4 Hormonal memory and habituation under repeated mechanical stimulation in sorghum stems

The experimental apparatus applied cyclic bending to sorghum stems for 2 h, followed by a 6 h resting period, repeated throughout the experiment. Hormone levels were examined at three time points: 1 h, 7 h, and 9 h AIMS, corresponding to 1 h after initiating the first round of mechanical stimulation, 1 h before the second round, and 1 h after initiating the second round, respectively. At 7 h AIMS, when stimulated plants had rested for 5 h after the first stimulation cycle, alterations in hormone levels were still detected, especially reduced GA_20_ and IAA levels across all four tissues, which may suggest a hormonal memory of prior stimulation. Similar transcriptional memory in response to mechanical stimulation has been reported in poplar, where 1145 genes were differentially expressed 30 min after a single mechanical stress event (Pomiès et al., 2017). Additionally, in *Arabidopsis*, 7 days of repetitive mechanical stimulation to juvenile plants triggered continuous upregulation of defense genes for up to 5 days after the mechanical stimulation ceased (Brenya et al., 2020). Similar stress memory mechanisms have also been reported under drought stress (Ding et al., 2012), hyper‐osmotic stress (Sani et al., 2013), and heat stress (Lämke et al., 2016).

In contrast, at 9 h AIMS, the same point in the second stimulation cycle as 1 h AIMS, the hormonal response was not identical to that observed at 1 h AIMS. Notably, GA_20_ and ABA levels no longer showed significant differences between control and stimulated tissues at 9 h AIMS, which may suggest desensitization or adaptation of sorghum stems to the second round of mechanical stimulation. Similar attenuation of molecular responsiveness has also been reported before. In poplar, a significant reduction in transcriptional response was observed as soon as the second bending was applied (Martin et al., 2010). In *Arabidopsis*, the induction of the ethylene biosynthesis gene *ACS6* was reduced following subsequent touch stimulation (Arteca and Arteca, 1999), and genes encoding the mitochondrial outer membrane protein also exhibited a desensitized transcriptional response after repetitive touch treatments, with the strongest response detected for the initial stimulus (Xu et al., 2019). Such desensitization or habituation is not limited to thigmomorphogenesis. It has also been detected in response to cold stress (Zarka et al., 2003), and is exemplified by the behavioral learning in *Mimosa pudica*, which eventually ceased leaf closure after prolonged repetitive mechanical stimulation (Gagliano et al., 2014). Taken together, these findings may suggest that both memory and habituation mechanisms operate in plants to optimize their response to mechanical stimuli. The memory system enables fine-tuned reactions based on prior exposure, and the habituation prevents excessive or energetically costly responses to repetitive and non-threatening mechanical stimuli.

## 5 Conclusions

Distinct stem tissues, including the pulvinus, white band, zone of division, and zone of maturation, exhibited differential basal levels of JA, ABA, IAA, and GA_1_ in control plants, which may indicate their unique contributions to stem morphogenesis. Upon mechanical stimulation, these tissues displayed both unique and shared hormonal responses. The JA level was specifically altered in the pulvinus, and the GA_1_ level was changed only in the zone of division. In contrast, reductions in GA_20_, IAA, and ABA levels were observed in all these tissues. These tissue-specific variations and shared responses may reflect both functional specialization and coordinated regulation among these stem tissues in response to mechanical stimulation. Mechanical stimulation resulted in shorter plants, likely due to a reduction in both internode number and cell elongation. Additionally, increased density and radial length of pith vascular bundles, enhanced lignification levels in both the pith and rind, and greater rind thickness were also observed following mechanical stimulation, which may collectively explain the increased strength and flexibility of mechanically stimulated sorghum stems reported in earlier studies.

The current study focused specifically on the cultivar “Della” grown under a 14 h light/10 h dark photoperiod. Future studies could investigate how diverse genotypes respond to mechanical stimulation under varying photoperiods, particularly given that sorghum is originally a short-day species. Such work will further enhance our understanding of thigmomorphogenesis and facilitate the breeding of lodging-resistant sorghum.

## Data Availability

The raw data supporting the conclusions of this article will be made available by the authors, without undue reservation.
