# Supplementary material for: Bioenergy sorghum stems respond to mechanical stimulation with tissue-specific modifications in hormone homeostasis and anatomical traits
Source: Front Plant Sci. 2026 Mar 12;17:1769393. doi: 10.3389/fpls.2026.1769393 (PMC13019638; doi:10.3389/fpls.2026.1769393)
Supplement: Supplementary file 1 [file Table1.docx]

**Bioenergy sorghum stems respond to mechanical stimulation with tissue-specific modifications in hormone homeostasis and anatomical traits**

**Qing Li^1,2, *^, Omid Zargar^3^, Sungkyu Park^2^, Matt Pharr^3^, Anastasia Muliana^3^, Tesfamichael H. Kebrom^1^, Scott A. Finlayson^2^**

^1^ Cooperative Agricultural Research Center, Prairie View A&M University, USA

^2^ Department of Soil and Crop Sciences, Texas A&M University, USA

^3^Department of Mechanical Engineering, Texas A&M University, USA

**^*^Corresponding author:** qili@pvamu.edu


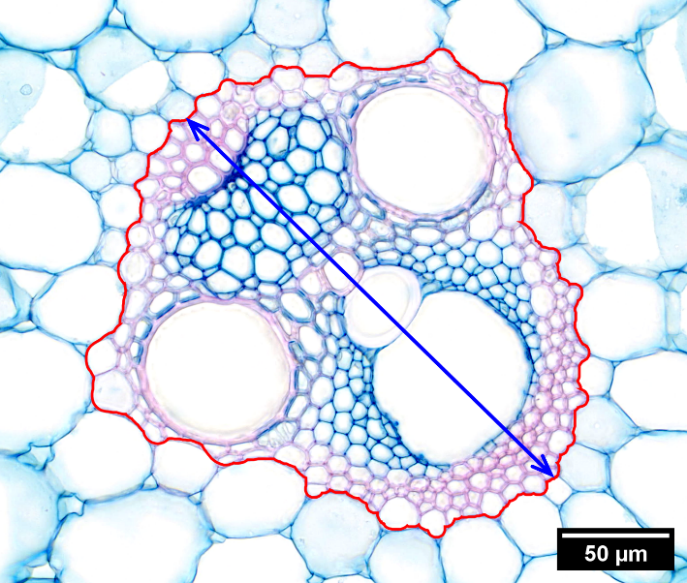


**Supplementary Fig. 1.** A Vascular Bundle (VB) in the pith of the stem. The red outline indicates the total VB area (size), and the blue line marks the VB radial length.


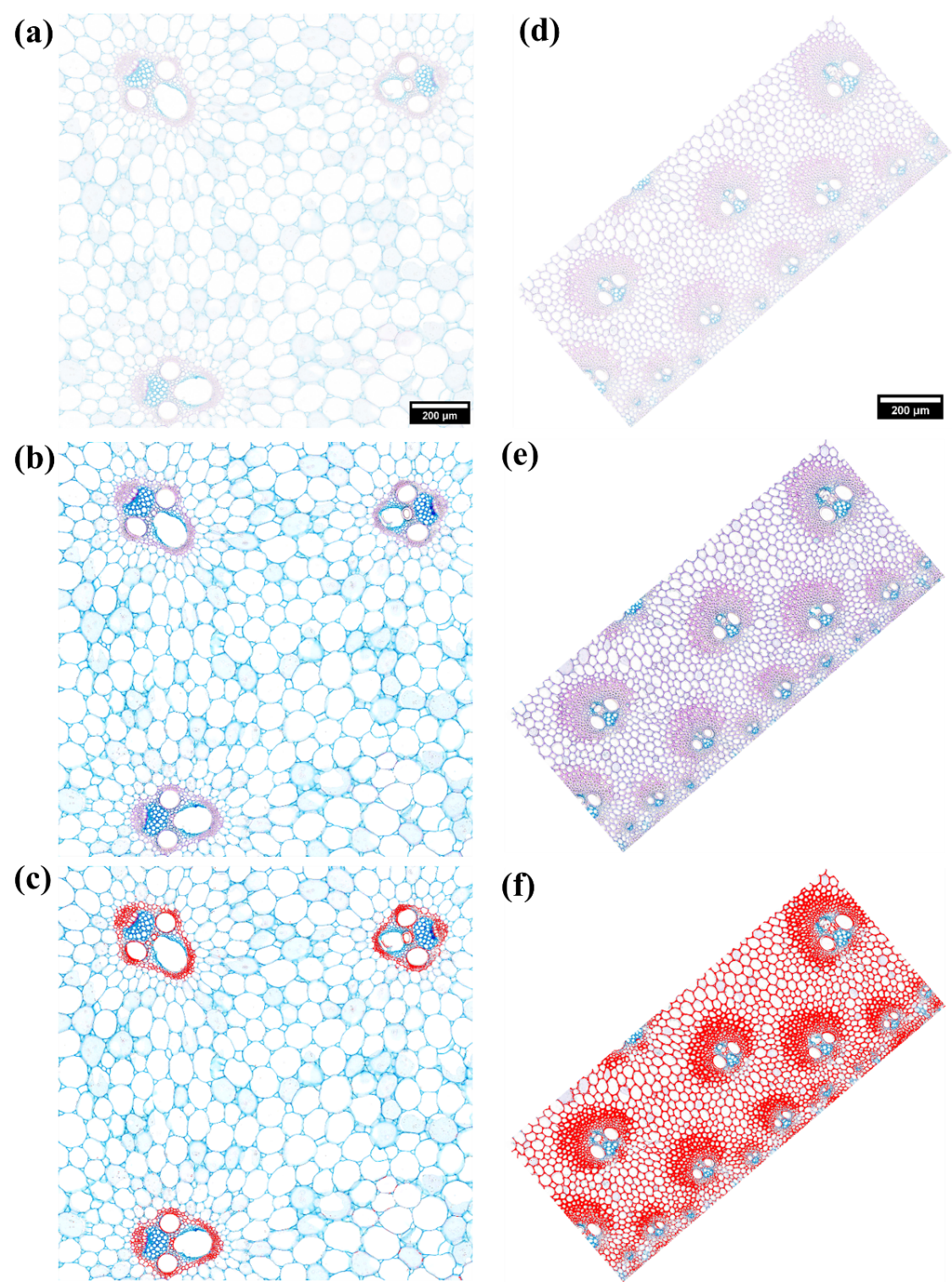


**Supplementary Fig. 2.** Segmentation of lignified regions in selected pith and rind areas of a stem cross-section image. Original images of the selected pith (**a**) and rind (**d)**. Corresponding images after brightness/contrast adjustment (**b, e**). Segmentation of lignified area in the pith (**c**) and rind (**f)**, with lignified tissue shown in red.


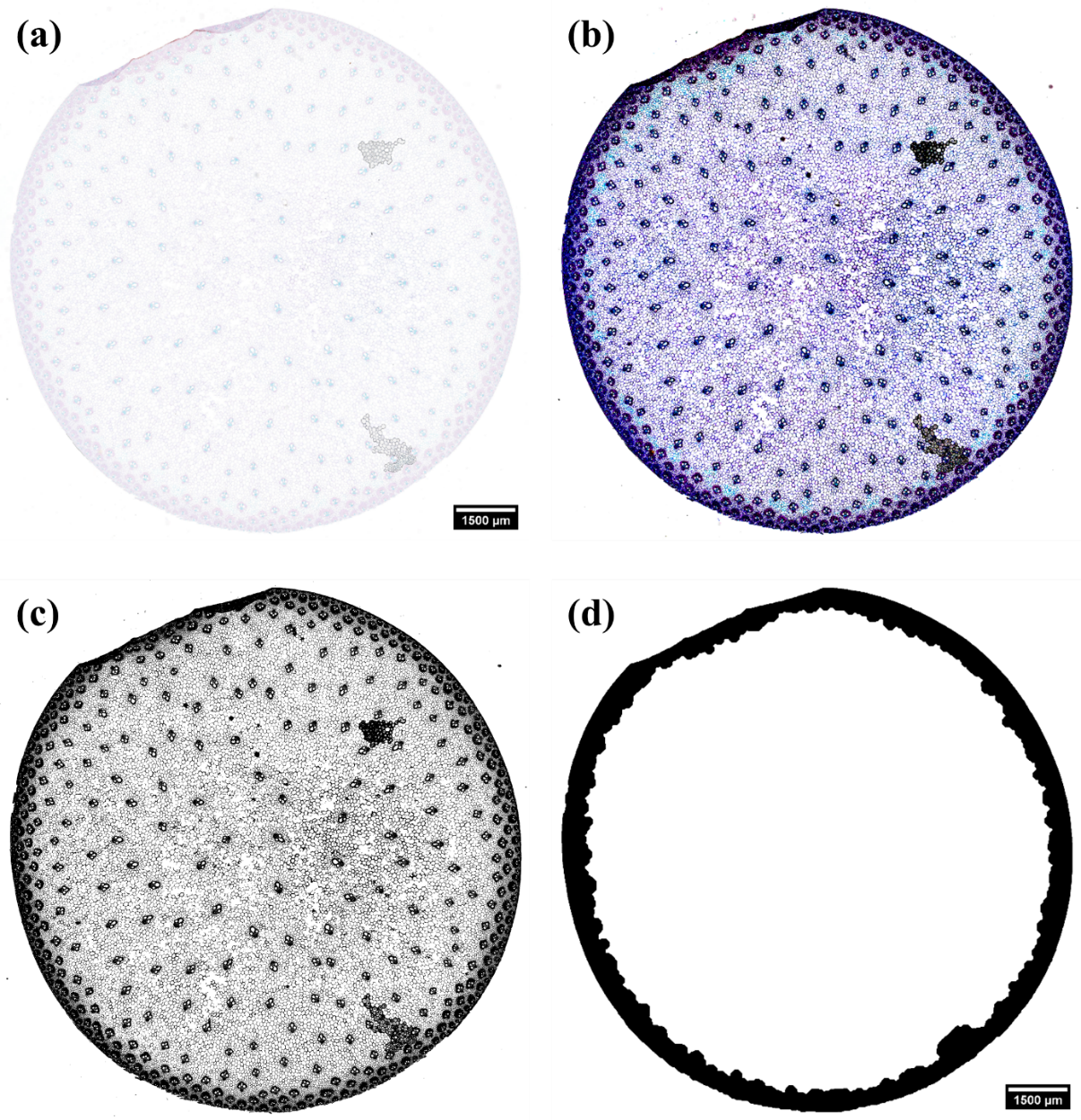


**Supplementary Fig. 3.** Segmentation of the rind in a stem cross-section image. **(a)** Downsampled whole-slide image. **(b)** Corresponding image after brightness/contrast adjustment. **(c)** Binary-converted image. **(d)** Rind segmentation.
